# Geodermatophilus maliterrae sp. nov., a member of the Geodermatophilaceae isolated from badland surfaces in the Red Desert, Wyoming, USA

**DOI:** 10.1099/ijsem.0.006603

**Published:** 2024-12-13

**Authors:** Seifeddine Ben Tekaya, Imen Nouioui, Gabryelle May Flores, Meina Neumann-Schaal, Felix Bredoire, Franco Basile, Linda T. A. van Diepen, Naomi L. Ward

**Affiliations:** 1 Department of Molecular Biology, University of Wyoming, 1000 E. University Avenue, Laramie, WY 82071, USA; 2 Leibniz Institute, DSMZ-German Collection of Microorganisms and Cell Cultures, Berlin, Germany; 3 Department of Botany, University of Wyoming, 1000 E. University Avenue, Laramie, WY 82071, USA; 4 Braunschweig Integrated Centre of Systems Biology (BRICS), Rebenring 56, 38106 Braunschweig, Germany; 5 Department of Chemistry, University of Wyoming, 1000 E. University Avenue, Laramie, WY 82071, USA; 6 Department of Ecosystem Science & Management, University of Wyoming, 1000 E. University Avenue, Laramie, WY 82071, USA; 7 Department of Microbiology, Immunology, and Pathology, Colorado State University, Fort Collins, USA

**Keywords:** *Geodermatophilaceae*, phylogenetic analysis, phylogenomic analysis, polyphasic taxonomy, Wyoming badland formation

## Abstract

A novel Gram-stain-positive, black-pigmented bacterium, designated as WL48A ^T^, was isolated from the surface of badland sedimentary rock in the Red Desert of Wyoming and characterized using a polyphasic taxonomic approach. Good growth occurred at 28–32 °C, pH 7–9, and NaCl less than 1% (w/v). Colonies, growing well on International Streptomyces Project media (ISP) 3 and ISP 7, were black and adhering to the agar. Phylogenetic analyses based on 16S rRNA gene and draft genome sequences showed that strain WL48A^T^ belongs to the family *Geodermatophilaceae*, forming a distinct sub-branch with *Geodermatophilus bullaregiensis* DSM 46841^T^. The organism showed 16S rRNA gene sequence similarity of 98.8% with *G. bullaregiensis* DSM 46841^T^. Digital DNA–DNA hybridization value between the genome sequences of strain WL48A ^T^ and *G. bullaregiensis* DSM 46841^T^ was 51.8%, below the threshold of 70% for prokaryotic species delineation. The chemotaxonomic investigation revealed the presence of galactose, glucose, mannose, xylose and ribose as well as *meso*-DAP in the peptidoglycan layer. The polar lipid profiles contained phosphatidylcholine (PC), phosphatidylinositol (PI), diphosphatidylglycerol (DPG), phosphatidylethanolamine (PE) phosphoglycolipid, phospholipids and an unidentified lipid. The menaquinone profile consisted of MK-9(H_4_) (98.2%) and MK-9(H_2_) (10.8%). The major fatty acid profile (>15%) comprised iso-C_15 : 0_ and iso-C_16 : 0_. Based on phenotypic, genetic and genomic data, strain WL48A^T^ (=DSM 116197^T^ = NCIMB 15483^T^=NCCB 100957^T^ =ATCC TSD-376^T^) merits to be considered as a novel species for which the name *Geodermatophilus maliterrae* sp. nov. is proposed.

## Introduction

Luedmann (1968) proposed the genus *Geodermatophilus*, which belongs to the family *Geodermatophilaceae*, which was first introduced by Normand *et al*. [1,3], then emended by Zhi *et al*. (2009) and Montero-Calasanz *et al.* (2017) [4,5]. The family traditionally encompassed four genera that included *Geodermatophilus* as the type genus [1], *Modestobacter*, *Blastococcus* and *Klenkia* [6]. Recently, additional genera have been amended, which are *Pleomorpha*, later corrected to *Petropleomorpha* [7] and *Trujillonella*, respectively [6]. According to the List of Prokaryotic names with Standings in Nomenclatures (https://lpsn.dsmz.de/genus/geodermatophilus), there are 20 species with validly published and correct names within the genus. The genus belongs to the order *Geodermatophilales* [8] and phylum *Actinomycetota* [9] and is characterized by positive Gram-reaction stain, aerobic growth and presence of rudimentary hyphae that develop into complex sporangia. Chemotaxonomically, MK-9(H_4_) is the predominant menaquinone, and *meso*-diaminopimelic acid (DAP) is prominent in the cell wall peptidoglycan [10,12]. Members of the genus have been isolated from a panoply of harsh habitats, which include rock surfaces [10,13,16] and arid soils [17,23], and, to a lesser extent, due to isolation difficulties, from various substrates such as fertile soils [24], lake sediments [25] and even marine sponge [12]. The genus *Geodermatophilus* possesses a genome size ranging from 4.7 to 5.8 Mb, with relatively high G+C content usually within the range of 73–75% [5]. In this paper, strain WL48A^T^ isolated from the surface of badland formations located in the Red Desert of Wyoming State, USA, was subjected to a polyphasic taxonomic description and found to form a type strain for a novel species for which the name *Geodermatophilus maliterrae* sp. nov. was proposed.

## Isolation and maintenance

Strain WL48A^T^ was isolated from the surface of a badland sedimentary rock located in the Red Desert of Wyoming (41.33028–107.77460). Deteriorated particles were collected from the upper 8–10 mm of the rock surface using a sterile rock hammer directly into a sterile Whirlpak bag. Serial dilutions using 1× PBS were performed, and aliquots of 100 µl from each dilution were plated onto GYM (glucose–yeast extract–malt) Streptomyces medium (DSMZ medium 65), Gauze’s No.1 medium (DSMZ medium 1048) [26] and *Geodermatophilus* (Geo)-medium (DSMZ medium 714). After ~3 weeks of incubation at 28 °C, an opaque, dry and elevated black colony was isolated from Geo-medium and subjected to purification through subculturing onto GYM Streptomyces agar medium. Pure cultures were preserved for long-term storage in glycerol stocks (25% w/v) at −80 ˚C. The strain WL48A^T^ has been deposited at the American Type Culture Collection, the Leibniz Institute DSMZ–German Collection of Microorganisms (DSMZ), The National Collection of Industrial, Food, and Marine Bacteria in the United Kingdom (NCIMB) and the Collection of Bacteria NCCB at the Westerdijk Fungal Biodiversity Institute in the Netherlands under accession numbers ATCC TSD-376 ^T^, DSM 116197 ^T^, NCIMB 15483 ^T^ and NCCB 100957^T^, respectively. The type strain *G. bullaregiensis* DSM 46841^T^, being the closest relative to the isolate WL48A^T^, was obtained from DSMZ-German Collection of Microorganisms, to serve as a comparative reference.

## Growth, morphological and physiological characteristics

A culture suspension grown on GYM medium for 7 days was washed with 1× PBS buffer and adjusted to a turbidity equivalent to ~5 McFarland units. This suspension was used as the inoculum. For media and temperature tests conducted in standard 90 mm Petri dishes, 250 µl of inoculum was used. For tests involving NaCl, pH and carbon sources, 12-well plates were utilized, and the inoculum was adjusted to 100 µl per well. All tests were performed in duplicate. The cultural and morphological features of the strain WL48A^T^ were determined after cultivation for 7 days at 28 °C using several growth media. These included GYM, *Geodermatophilus* (Geo), Gause No1, Luedmann and International Streptomyces Project (ISP) 1 to 7 media. The growth temperature range of the strain was determined on GYM medium plates, with temperatures spanning from 15 to 45 °C, incubated for 7 days. To assess tolerance to pH (4 to 10, with an increment of 1), the strain was grown on ISP2 media adjusted to the target pH value using either an HCl or NaOH solution. Similarly, strain WL48A^T^ response to various concentrations of NaCl (0, 0.5%, 1–15% with an increment of 1) was assessed by growth on ISP2 media supplemented with the target concentration and grown for 7 days at 28 °C. In addition, the Gram stain reaction was performed [27], and the cell morphology was described using an optical microscope (OLYMPUS BH2).

The inoculum for the API tests was prepared according to the manufacturer’s recommendations. Specifically, a GYM plate was streaked and incubated for 7 days at 28 °C. The resulting colonies were then harvested and homogenized in the provided suspension solution to achieve the required density for each test as specified by the manufacturer. Physiological features such as catalase, nitrate reduction, urease, citrate and hydrogen sulfide (H_2_S) utilization, degradation of gelatin and indole production were determined using API CORYNE and API 20E (bioMérieux) strips, and duplicates were used for both WL48A^T^ and *G. bullaregiensis* DSM 46841^T^. In addition, the enzymatic profile of the studied strains was determined using API ZYM strips (bioMérieux). These tests were performed for both WL48A^T^ and *G. bullaregiensis* DSM 46841^T^. Growth on various carbon sources was assessed using basal mineral salt agar medium (per litre of deionized water: (NH_4_)_2_SO_4_ 2.64 g l^−1^, KH_2_PO_4_ 2.38 g l^−1^, K_2_HPO_4_ 4.31 g l^−1^, MgSO_4_.7 H_2_O 1 g l^−1^ and 1 ml l^−1^ trace element solution, 15 g agar) amended with 1% (w/v) of arabinose, fructose, galactose, inositol, mannitol, raffinose, rhamnose, sucrose and xylose. The positive control consisted of the basal medium added with 1% glucose, while the negative control consisted of the basal medium alone [28].

The strain grew well on GYM agar, Luedmann, Geo-medium, Gauze No.1 medium, ISP2 and ISP4. ISP3 and ISP7 exhibited the fastest (~4 days) and most remarkable growth, with dry black colonies exhibiting irregular surfaces. Growth on ISP5 was weak with a faint black colour. No growth was observed on ISP1 or ISP6. Under an optical microscope (OLYMPUS BH2), Gram-positive cells appeared pleomorphic, with shapes ranging from spherical to almost cuboid, with the presence of rod-shaped cells, concurrent with previous descriptions of *Geodermatophilus* morphology [1,15, 29].

Growth of strain WL48A^T^ was observed at 28–32 °C (optimal growth at 30 °C) and the pH range from 7 to 9 (optimum7.5). The strain was found to grow in the presence of less than 1% NaCl but not 1% or higher.

Strain WL48A^T^ exhibited a negative reaction for nitrate reduction, indole production, acetoine production, H_2_S production, use of urease, citrate and catalase reaction. It also tested negative for esterase, esterase lipase, lipase, cysteine arylamidase, trypsin, acid phosphatase, naphthol-AS-BI-phosphohydrolase, α-galactosidase, β-galactosidase, β-glucuronidase, β-glucosidase, *N-*acetyl-β-glucosaminidase, α-mannosidase and α-fucosidase. The strain was, however, capable of hydrolysing gelatin. It also exhibited a positive reaction for alkaline phosphatase, leucine arylamidase, valine arylamidase, α-chymotrypsin and α-glucosidase. The strain grew in the presence of sole carbon sources such as glucose, fructose, galactose, myo-inositol, raffinose, rhamnose, sucrose and xylose but did not grow on arabinose and mannitol. Table 1 contrasts the phenotypic and physiological characteristics of strain WL48A^T^ as compared to its closest relative *G. bullaregiensis* DSM 46841^T^.

**Table 1.** Differential characteristics of strain WL48A^T^ and its closest relative type strain *G. bullaregiensis* DSM 46841^T^

Characteristics	WL48A^T^	*G. bullaregiensis* DSM 46841**^T^**
Colony colour on GYM	Black	Black greenish
Colony surface on GYM	Dry	Dry
pH optimum	7–9	7–8.5
Temperature optimum (°C)	28–30	25–35
**Utilization of**		
Arabinose	−	**^+^**
Myo-inositol	**^+^**	−
**Enzymes**		
Esterase	−	**^+^**
Esterase lipase	−	**^+^**
Cysteine arylamidase	−	**^+^**
Trypsin	−	**^+^**
Acid phosphatase	−	**^+^**
β-Glucosidase	−	**^+^**
Gelatin hydrolysis	**^+^**	−
Catalase	−	**^+^**

+: presence, –: absence. Both strains were positive for glucose, fructose, galactose, raffinose, rhamnose, sucrose, xylose, alkaline phosphatase, leucine arylamidase, valine arylamidase, α-chymotrypsin, α-glucosidase and gelatin hydrolysis. Both strains were negative for mannitol, lipase, naphtol-AS-BI-phosphohydrolase, α-galactosidase, β-galactosidase, β-glucuronidase, *N-*acetyl-β-glucosaminidase, α-mannosidase, α-fucosidase and catalase.

## 16S rRNA gene phylogeny

**Fig. 1.**
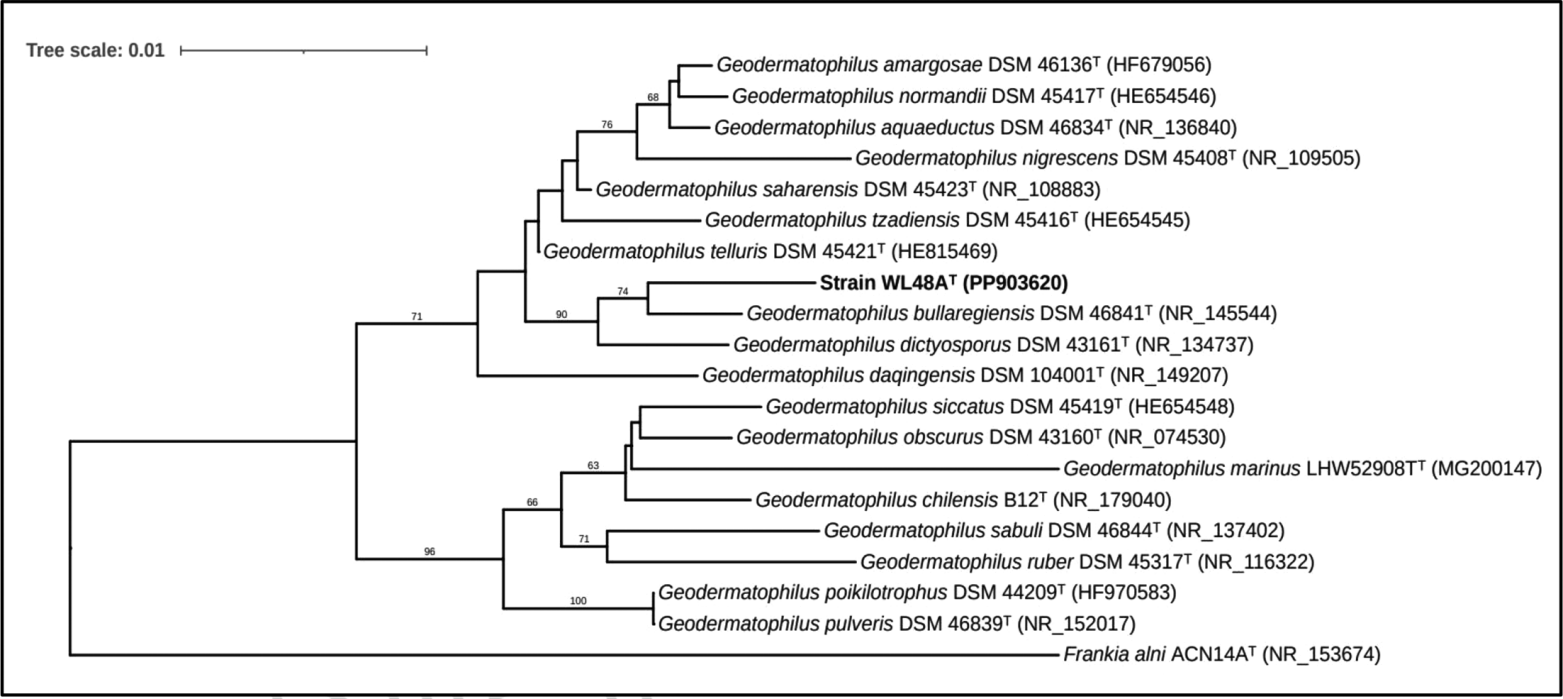
Tree inferred with FastME 2.1.6.1 from GBDP distances calculated from 16S rRNA gene sequences. The branch lengths are scaled in terms of the GBDP distance formula d5. The numbers above branches are GBDP pseudo-bootstrap support values>60% from 100 replications. The tree was rooted at the midpoint. *Frankia alni* ACN14A^T^ was used as an outgroup.

Strain WL48A^T^ was cultivated in Geo-medium broth (DSMZ medium 714) shaken at 150 r.p.m. for 7 days at 28 °C; genomic DNA was extracted using a commercial genomic DNA extraction kit (Qiagen). Amplification of the 16S rRNA gene was done using universal bacterial primer sets, 27F (5′-GAGTTTGATCCTGGCTCAG-3′) and 1525R (5′-AGAAAGGAGGTGATCCAGCC-3′) as described by Rainey *et al*. [30]. PCR products were purified using EXOSAP-IT Express (Applied Biosystems), and sequences were processed at Eurofins Genomics via SANGER sequencing technology. The 16S rRNA sequence of strain WL48A^T^ was analysed using Geneious Prime (version 2022.2.2), and the identity of the consensus sequence (1450 bp) was compared to the full-length 16S rRNA sequence (1559 bp) retrieved from the full genome sequence. Pairwise sequence similarities were calculated as recommended by Meier-Kolthoff *et al*., for the 16S rRNA gene [31]. Phylogenies were inferred by the GGDC (Genome-to-Genome Distance Calculator) web server [32] available at  http://ggdc.dsmz.de/ using the DSMZ phylogenomics pipeline adapted to single genes [33]. A multiple sequence alignment was created with muscle [34], and a balanced minimum evolution tree was inferred via FastME 2.1.6.1 [35]. In parallel, maximum-likelihood [36], neighbour-joining [37] and maximum-parsimony [38] trees were also generated using MEGAX [39]. A phylogenetic analysis targeting the 16S rRNA gene sequence classified the isolate WL48A^T^ within the genus *Geodermatophilus* (Figs 1 and S1, available in the online version of this article), with *G. bullaregiensis* DSM 46841^T^ sharing the closest similarity of 98.8% (Table 2).

**Table 2.** Comparative analysis of 16S rRNA similarities, dDDH and ANI values for WL48A^T^ with *Geodermatophilus* type strains

WL48A^T^	16S rRNA	dDDH (%) [CI]*	ANI (%)
*Geodermatophilus bullaregiensis* DSM 46841^T^	98.8	51.8 [49.1–54.5]	93.3
*Geodermatophilus tzadiensis* DSM 45416 ^T^	98.1	31.6 [29.2–34.1]	86.5
*Geodermatophilus telluris* DSM 45421 ^T^	97.3	31.2 [28.8–33.7]	86.2
*Geodermatophilus amargosae* DSM 46136 ^T^	98.1	30.1 [27.7–32.6]	85.7
*Geodermatophilus normandii* DSM 45417 ^T^	98.2	29.8 [27.4–32.3]	85.5
*Geodermatophilus aquaeductus* DSM 46834 ^T^	98.2	29.6 [27.2–32.1]	85.5
*Geodermatophilus saharensis* DSM 45423 ^T^	98.2	29.3 [26.9–31.8]	85.1
*Geodermatophilus dictyosporus* DSM 43161 ^T^	98.0	29.1 [26.7–31.6]	86.5
*Geodermatophilus nigrescens* DSM 45408 ^T^	97.7	28.7 [26.4–31.2]	84.9
*Geodermatophilus obscurus* DSM 43160 ^T^	97.6	26.5 [24.2–29.0]	82.6
*Geodermatophilus siccatus* DSM 45419 ^T^	96.9	26.4 [24.1–28.9]	83.0
*Geodermatophilus poikilotrophus* DSM 44209 ^T^	97.3	26.4 [24.0–28.9]	82.8
*Geodermatophilus chilensis* B12^T^	96.7	26.4 [24.0–28.8]	82.9
*Geodermatophilus pulveris* DSM 46839 ^T^	97.5	25.4 [23.1–27.9]	82.3
*Geodermatophilus sabuli* DSM 46844 ^T^	96.0	25.0 [22.7–27.5]	83.0
*Geodermatophilus ruber* DSM 45317 ^T^	96.1	25.0 [22.7–27.5]	81.8
*Geodermatophilus marinus* LHW52908^T^	96.4	24.7 [22.4–27.2]	81.6
*Geodermatophilus daqingensis* DSM 104001 ^T^	98.1	23.9 [21.6–26.4]	80.3

Exhibited dDDH values are from *d4* formula.

*[CI]: confidence interval.

## Overall genomic relatedness and phylogenomics

Full genome sequencing was performed at SeqCenter LLC, Pennsylvania. In brief, sample libraries were prepared using the Illumina DNA Prep kit and sequenced on an Illumina NovaSeq 6000, producing paired-end 151 bp reads. Demultiplexing, quality control and adapter trimming were performed with bcl-convert (v4.1.5). The draft genome of strain WL48A^T^ has been submitted to GenBank with the accession JBFNXQ000000000. The genome assemblies’ parameters were as follows: (i) genome size of 5.4 Mb, (ii) G+C content of 74.7%, (iii) scaffold counts of 250, (iv) N50 lengths of 44113 and (v) sequencing coverage of ~248. The genome size of the closest relative *G. bullaregiensis* DSM 46841^T^ is 5.2 Mb, and the G+C content is 74.9%. BUSCO v5.4.3 was used to assess genome completeness, which returned a result of 98.4%.

Taxogenomic analysis was performed using the Type Strain Genome Server (https://tygs.dsmz.de/) [40] and closely related type strains retrieved from the List of Prokaryotic Names with Standing in Nomenclature (https://lpsn.dsmz.de) [32]. A pairwise comparison between the genome sequence of strain WL48A^T^ and closely related type strains was estimated, and intergenomic distances were inferred based on algorithm trimming and d5 distance formula, with a total of 100 resampling replicates [35]. An additional phylogenomic tree based on single-copy genes was also reconstructed based on 589 single-copy genes [41]. Digital DNA–DNA hybridization (dDDH) values and confidence intervals were calculated using the recommended settings of the GGDC version 4.0 [42]. Average nucleotide identity (ANI) values were calculated between strain WL48A^T^ and other *Geodermatophilus* species using the Ez-ANI tool (www.ezbiocloud.net/tools/ani) [43].

**Fig. 2.**
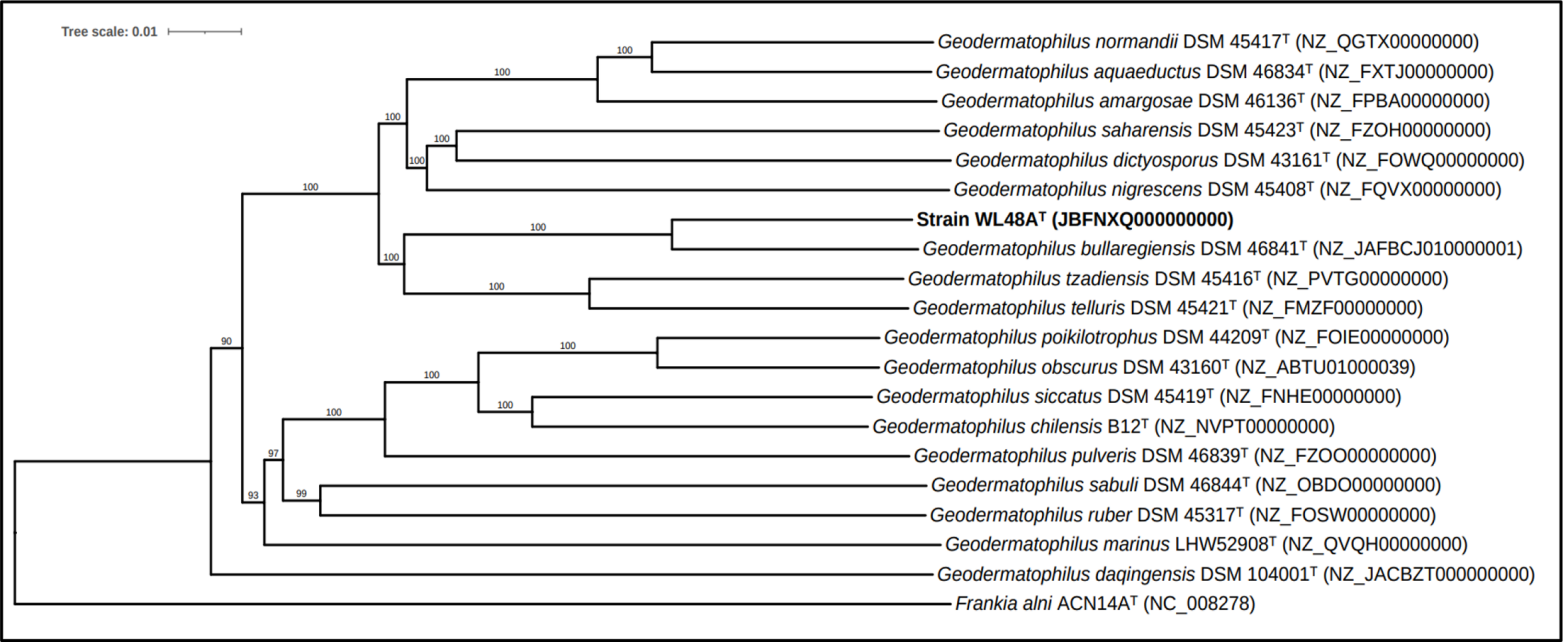
Phylogenomic tree reconstructed using FastME 2.1.6.1 based on GBDP distances inferred from the genome sequences of strain WL48A^T^ and closely related *Geodermatophilus* type strains. The branch lengths on the tree are proportional to the GBDP distance calculated using the d5 formula. Pseudo-bootstrap support values are depicted above the branches, all exceeding 60% across 100 replications. The tree was rooted at its midpoint. *Frankia alni* ACN14A was used as an outgroup. The scale bar indicates evolutionary distance (substitutions per position).

All the dDDH values between the genomic sequence of the strain and the type strains of the genus *Geodermatophilus* were below the 70% threshold recommended for species delineation [44]. The values ranged from 51.8 to 19.3% with the highest scoring to the type strain *G. bullaregiensis* DSM 46841^T^. The ANI value between WL48A^T^ and the closest relative type species *G. bullaregiensis* DSM 46841^T^ was 93.31%, which is below the threshold of 95–96% employed to delineate different species [45]. Table 2 summarizes dDDH, ANI and 16S rRNA sequence similarity for strain WL48A^T^ and its closely related *Geodermatophilus* type strains. Additionally, the phylogenomic trees show that strain WL48A^T^ forms a well-supported distinct subclade with the type strain of *G. bullaregiensis* species (Figs 2 and S2).

## Extremotolerance and secondary metabolites

Members of the genus *Geodermatophilus* are capable of withstanding a range of abiotic stresses. Rainey *et al*. and Gtari *et al*. demonstrated the extraordinary capability of *Geodermatophilus obscurus* to cope with stressors such as desiccation, heavy metals and radiation [46,47]. The levels of radiation resistance of the strain reported in that study (both gamma and UV) were particularly high, standing in doses close to those observed in the model extremophile *Deinococcus radiodurans* [48]. The proteogenome of *G. obscurus* revealed robust radiation resistance mechanisms, based essentially on a complex DNA repair machinery [49]. In the current study, in order to gain a primordial understanding on the adaptive capabilities of strain WL48A^T^, a comparative genomic analysis was conducted with the closest relative *G. bullaregiensis* DSM 46841^T^. The Rapid Annotation Subsystem Technology server RAST was used to generate an annotation file, which was then fed into the seed server to make an inference on key gene distribution [50,51]. Strains WL48A^T^ and DSM 46841^T^ exhibited identical profiles for DNA repair mechanisms, suggesting a conserved reparation machinery linked to high UV exposure, i.e. the surface of sedimentary rock in the Wyoming Red Desert and the surface of a monument edifice with prolonged sun exposure in the Bulla Regia region of Tunisia. In addition, both strains harboured genes that are necessary for coping with oxidative stress and carbon starvation, as well as bacterial haemoglobins and sigmaB. Furthermore, with relation to osmotic stress, both strains displayed genes involved in choline and betaine uptake and betaine biosynthesis. These results are in line with recent observations on the genome of newly described *Blastococcus* [52], suggesting a possible conserved mechanism to cope with various osmotic stressors among *Geodermatophilaceae* members that thrive on rock surfaces. With regard to antibiotics and toxic compounds, both strains harboured similar profiles related to fluoroquinolones, beta-lactamase, cobalt-zinc-cadmium resistance, mercury and mercuric reductase and resistance to chromium compounds. Both strains showcased a particularly strong activity related to copper homeostasis, hinting to adaptive strategies to cope with high copper concentrations that could be encountered at the lithic interface [53]. Strain WL48A^T^ displayed, however, a unique subset of genes that confers resistance against arsenic, which include an arsenic pump-driving ATPase gene, an arsenate reductase gene, an arsenical-resistance protein ACR3-encoding gene and an arsenical resistance operon repressor. This feature might hint to an adaptation to specific stressors, e.g. arsenic deposits dispersed from the Yellowstone area, while *G. bullaregiensis* DSM 46841^T^ had a unique detoxification feature against selenite and selenate (Fig. S3).

Secondary metabolite-associated biosynthetic gene clusters (BGCs) were determined for both strains using antiSMASH (version 7.1.0) [54]. WL48A^T^ exhibited nine BGCs while *G. bullaregiensis* DSM 46841^T^ displayed six BGCs (Tables S1 and S2). Both strains synthesized isorenieratene and tetrocarcine A (terpene), loseolamycin A1/loseolamycin A2 (T3PKS), lancacydin C (redox-cofactor) and formicamycins A-M (T2PKS). However, unique to WL48A^T^ was the production of xantholipin (T2PKS) and maduropeptin (T1PKS), two antitumor compounds that were described within *Streptomyces flavogriseus* and *Actinomadura madurae*, respectively [55,56].

## Chemotaxonomy

Biomasses of strain WL48A^T^ were collected from a 7-day-old culture in a GYM medium shaken at 150 r.p.m. at 28 °C. The harvested biomasses were washed three times with sterile saline solution (0.8% NaCl) and then freeze-dried. Whole-cell sugars, DAP isomers of the peptidoglycan and polar lipids were determined using standard chromatographic procedures [57,59]. The menaquinone profile of the strain was identified following the protocol of Schumann *et al*. [60]. Fatty acids of the strain and its closest relative, strain DSM 46841^T^, were extracted from wet biomasses and identified as described by Sasser [61] and Vieira *et al.* [62].

Strain WL48A^T^ had galactose, glucose, mannose, xylose and ribose as well as *meso*-DAP in its peptidoglycan. The polar lipid profile contained phosphatidylcholine (PC), phosphatidylinositol (PI), diphosphatidylglycerol (DPG), phosphatidylethanolamine (PE), a phosphoglycolipid, phospholipids and an unidentified lipid (Fig. S4). The menaquinone profile of strain WL48A^T^ included MK-9(H_4_) (98.2%) and MK-9(H_2_) (10.8%). The major fatty acid profile (>15%) of the strain WL48A^T^ and strain DSM 46841^T^ comprised iso-C_15 : 0_ and iso-C_16 : 0_ (Table S3).

These results were coherent with the diagnostic sugars (galactose and glucose), *meso*-DAP, major polar lipids (PC, PI, PE, DPG), predominant menaquinone (MK-9(H_4_)) and major fatty acids (iso-C_15 : 0_, iso-C_16 : 0_) found in the type strain of * G. bullaregiensis* [10].

In summary, based on our polyphasic taxonomic approach, we confirm that strain WL48A^T^ exhibits significant differences to its closest relative type species, therefore justifying its proposal as the type strain for the novel species, for which the name * G. maliterrae* sp. nov. is proposed.

## Description of *Geodermatophilus maliterrae* sp. nov.

*Geodermatophilus maliterrae* (ma.li.ter'rae. L. masc. adj. *malus*, bad; L. fem. n. *terra*, land; N.L. gen. n. *maliterrae*, of badland, in reference to Wyoming badland sedimentary formations, origin of isolation of the type strain).

Strain is aerobic and forms black colonies with irregular dry surface. The cells are Gram-reaction-positive, catalase, indole and nitrate reduction negatives, spherical to cuboid and rod. It grows in the presence of less than 1% NaCl. Growth is observed at temperatures ranging from 28 to 32 ˚C with optimal growth at 30 ˚C, and across pH levels spanning from 7 to 9, with optimum being 7.5. Additionally, it is capable of hydrolysing gelatin, but not citrate, urease, acetoin or H_2_S. It utilizes glucose, fructose, galactose, myo-inositol, raffinose, rhamnose, sucrose and xylose but not arabinose or mannitol. The strain exhibits a positive reaction for alkaline phosphatase, leucine arylamidase, valine arylamidase, α-chymotrypsin and α-glucosidase but a negative reaction for esterase, esterase lipase, lipase, cysteine arylamidase, trypsin, acid phosphatase, naphthol-AS-BI-phosphohydrolase, α-galactosidase, β-galactosidase, β-glucuronidase, β-glucosidase, *N-*acetyl-β-glucosaminidase, α-mannosidase and α-fucosidase. The chemotaxonomic analysis revealed the presence of galactose, glucose, mannose, xylose and ribose as well as *meso*-DAP in the peptidoglycan layer. The polar lipid profiles contain PC, PI, DPG, PE, a phosphoglycolipid, phospholipids and an unidentified lipid. The menaquinone profile consists of MK-9(H_4_) (98.2%) and MK-9(H_2_) (10.8%). The main fatty acids consist of iso-C_15 : 0_, iso-C_16 : 0_, anteiso-C_17 : 0_, C_16 : 0_, C_18 : 0_, C_17 : 0_, iso-C_17 : 0_ and anteiso-C_15 : 0_.

The strain WL48A^T^ (=DSM 116197^T^ = NCIMB 15483^T^=NCCB 100957^T^ =ATCC TSD-376^T^) was isolated from a badland sedimentary rock formation in the Red Desert of Wyoming, USA. The draft genome of strain WL48A^T^ has a size of 5.4 Mbp, with a G+C content of 74.72%. The GenBank accession numbers for the 16S rRNA gene and draft genome sequences for strain WL48A^T^ are PP903620 and JBFNXQ000000000, respectively.

## Supplementary material

10.1099/ijsem.0.006603 Uncited Supplementary Material 1.

## Abbreviations

ANI: average nucleotide identity
BGCs: biosynthetic gene clusters
DAP: diaminopimelic acid
dDDH: digital DNA-DNA hybridization
DPG: diphosphatidylglycerol
GGDC: genome-to-genome distance calculator
GYM: glucose–yeast extract–malt extract
H_2_S: hydrogen sulfide
ISP: International Streptomyces Project
PC: phosphatidylcholine
PE: phosphatidylethanolamine
PI: phosphatidylinositol
