# Supplementary material for: Geodermatophilus maliterrae sp. nov., a member of the Geodermatophilaceae isolated from badland surfaces in the Red Desert, Wyoming, USA
Source: Int J Syst Evol Microbiol. 2024 Dec 13;74(12):006603. doi: 10.1099/ijsem.0.006603 (PMC12453565; doi:10.1099/ijsem.0.006603)
Supplement: Uncited Supplementary Material 1. [file ijsem-74-06603-s001.pdf]

Supplementary Materials

Geodermatophilus maliterrae sp. nov., a member of the *Geodermatophilaceae* isolated from badland surfaces in the Red Desert, Wyoming

Seifeddine Ben Tekaya[1], Imen Nouioui[2], Gabryelle May Flores[3], Meina Neumann-Schaal[2,4], Felix Bredoire[3], Franco Basile[5], Linda T.A. van Diepen[6] and Naomi L. Ward[7]

Author affiliations:

[1]Department of Molecular Biology, University of Wyoming, USA; University of Wyoming, 1000 E. University Avenue, Laramie, WY 82071

[2]Leibniz institute, DSMZ-German Collection of Microorganisms and Cell Cultures

[3]Department of Botany, University of Wyoming, USA; University of Wyoming, 1000 E University Avenue, Laramie, WY 82071

[4]Braunschweig Integrated Centre of Systems Biology (BRICS), Rebenring 56, 38106 Braunschweig, Germany

[5]Department of Chemistry, University of Wyoming, USA; University of Wyoming, 1000 E University Avenue, Laramie, WY 82071

[6]Department of Ecosystem Science & Management, University of Wyoming, USA; University of Wyoming, 1000 E University Avenue, Laramie, WY 82071

[7]Department of Microbiology, Immunology, and Pathology, Colorado State University, Fort Collins, USA

Correspondence: Seifeddine Ben Tekaya: sbenteka@uwyo.edu or sbenteka@gmail.com

Keywords: *Geodermatophilaceae*, Wyoming badlands formation, polyphasic taxonomy, phylogenetic analysis, phylogenomic analysis

Author notes: The GenBank accession numbers for the 16S rRNA gene and draft genome sequences for strain WL48A^T are PP903620 and JBFNXQ000000000, respectively.

Abbreviations: ANI, Average Nucleotide Identity; dDDH, digital DNA-DNA hybridization; GGDC, Genome-to-Genome Distance Calculator; GYM, glucose–yeast extract–malt extract; ISP, International Streptomyces Project

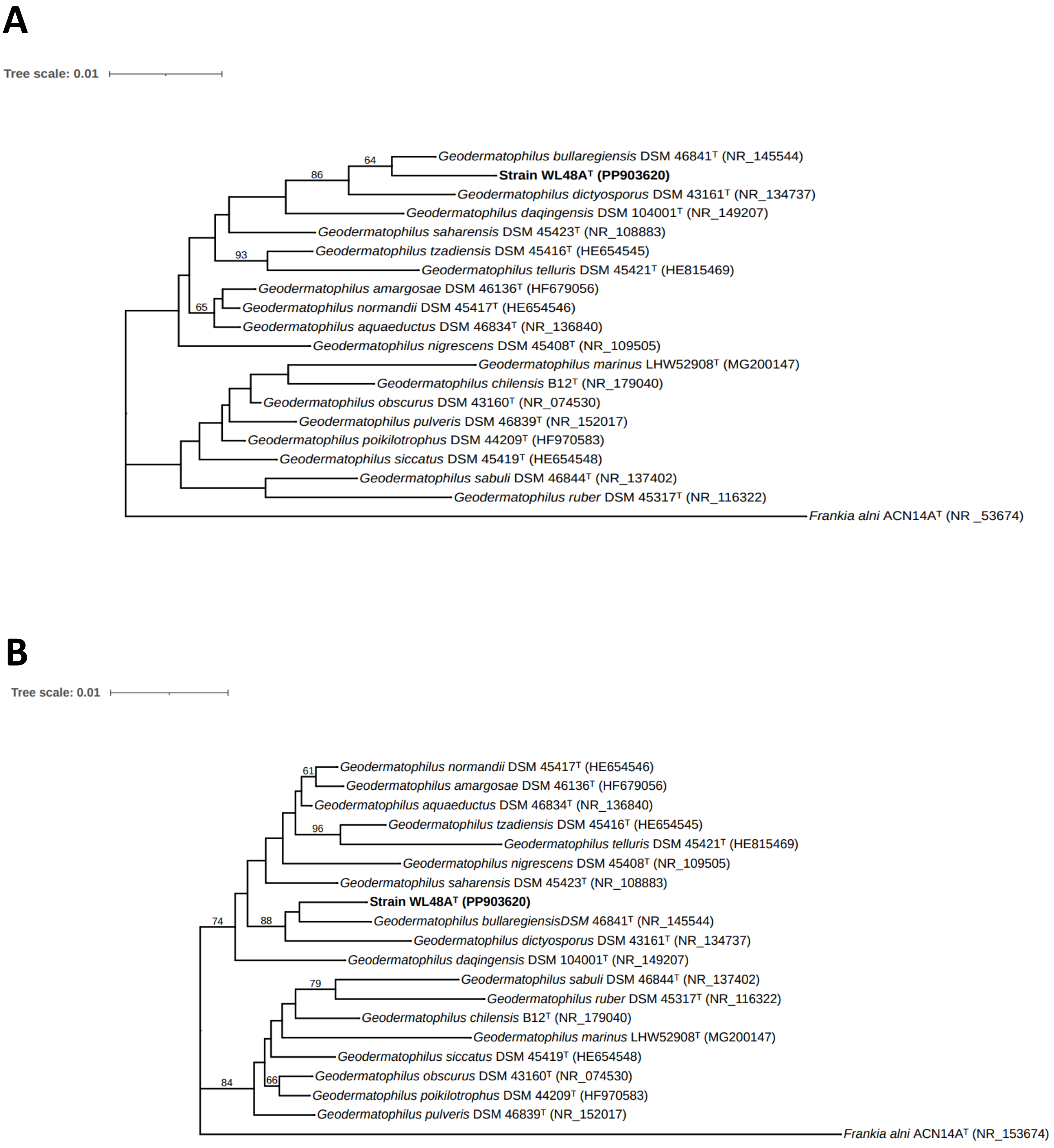
A
Tree scale: 0.01
64
86
93
65
Geodermatophilus bullaregiensis DSM 46841^{T} (NR_145544)
Strain WL48A^{T} (PP903620)
Geodermatophilus dictyosporus DSM 43161^{T} (NR_134737)
Geodermatophilus daqingensis DSM 104001^{T} (NR_149207)
Geodermatophilus saharensis DSM 45423^{T} (NR_108883)
Geodermatophilus tzadiensis DSM 45416^{T} (HE654545)
Geodermatophilus telluris DSM 45421^{T} (HE815469)
Geodermatophilus amargosae DSM 46136^{T} (HF679056)
Geodermatophilus normandii DSM 45417^{T} (HE654546)
Geodermatophilus aquaeductus DSM 46834^{T} (NR_136840)
Geodermatophilus nigrescens DSM 45408^{T} (NR_109505)
Geodermatophilus marinus LHW52908^{T} (MG200147)
Geodermatophilus chilensis B12^{T} (NR_179040)
Geodermatophilus obscurus DSM 43160^{T} (NR_074530)
Geodermatophilus pulveris DSM 46839^{T} (NR_152017)
Geodermatophilus poikilotrophus DSM 44209^{T} (HF970583)
Geodermatophilus siccatus DSM 45419^{T} (HE654548)
Geodermatophilus sabuli DSM 46844^{T} (NR_137402)
Geodermatophilus ruber DSM 45317^{T} (NR_116322)
Frankia alni ACN14A^{T} (NR _53674)
B
Tree scale: 0.01
61
96
74
88
79
84
66
Geodermatophilus normandii DSM 45417^{T} (HE654546)
Geodermatophilus amargosae DSM 46136^{T} (HF679056)
Geodermatophilus aquaeductus DSM 46834^{T} (NR_136840)
Geodermatophilus tzadiensis DSM 45416^{T} (HE654545)
Geodermatophilus telluris DSM 45421^{T} (HE815469)
Geodermatophilus nigrescens DSM 45408^{T} (NR_109505)
Geodermatophilus saharensis DSM 45423^{T} (NR_108883)
Strain WL48A^{T} (PP903620)
Geodermatophilus bullaregiensisDSM 46841^{T} (NR_145544)
Geodermatophilus dictyosporus DSM 43161^{T} (NR_134737)
Geodermatophilus daqingensis DSM 104001^{T} (NR_149207)
Geodermatophilus sabuli DSM 46844^{T} (NR_137402)
Geodermatophilus ruber DSM 45317^{T} (NR_116322)
Geodermatophilus chilensis B12^{T} (NR_179040)
Geodermatophilus marinus LHW52908^{T} (MG200147)
Geodermatophilus siccatus DSM 45419^{T} (HE654548)
Geodermatophilus obscurus DSM 43160^{T} (NR_074530)
Geodermatophilus poikilotrophus DSM 44209^{T} (HF970583)
Geodermatophilus pulveris DSM 46839^{T} (NR_152017)
Frankia alni ACN14A^{T} (NR_153674)

C

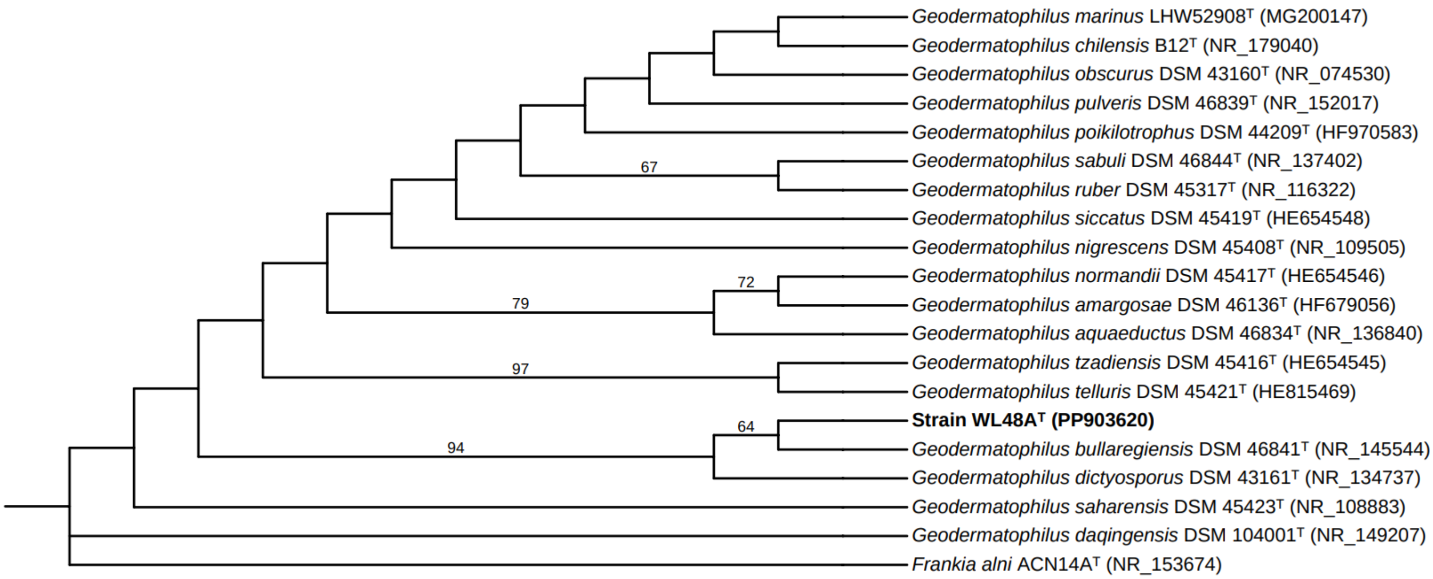

Figure S1. Maximum likelihood **(A)**, Neighbor joining **(B),** and Maximum parsimony **(C)** trees illustrating the positioning of strain WL48A^{T} within the genus *Geodermatophilus*. The phylogenetic trees were constructed using MEGAX software (version 10.2.5). The numbers above branches are bootstrap support values exceeding 60% across 100 replications. *Frankia alni* ACN14A^{T} was used as outgroup.

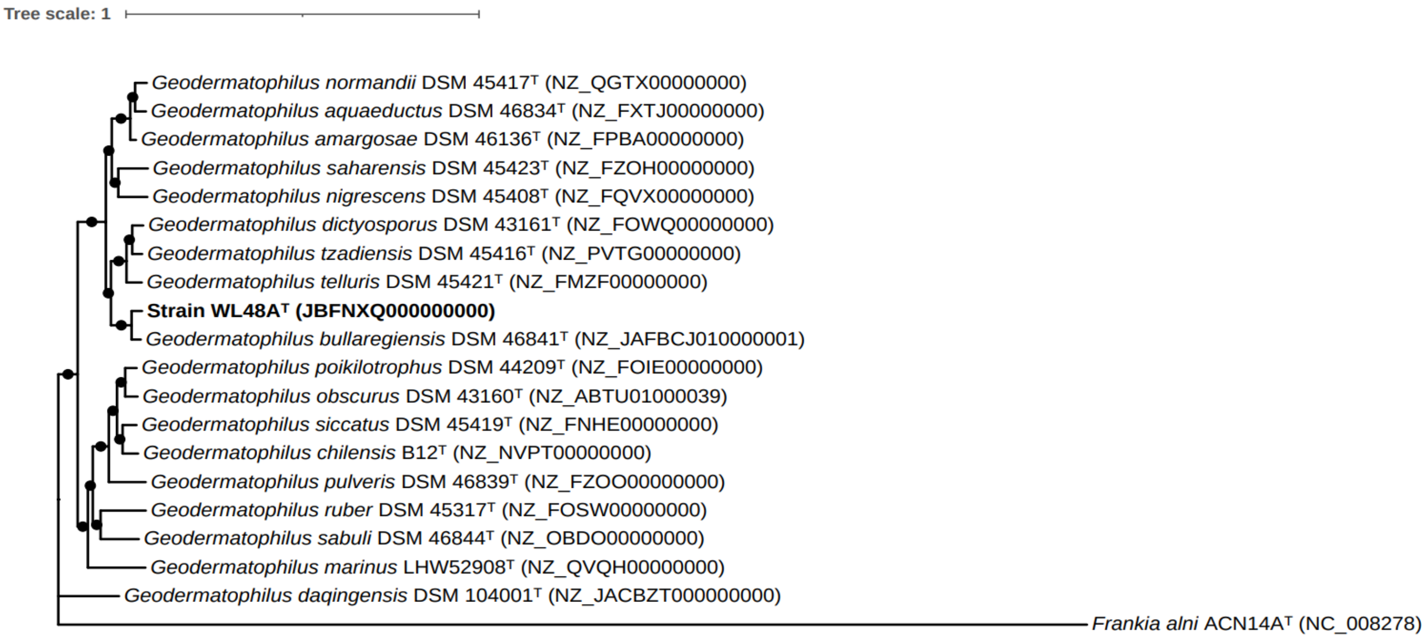

Figure S2. Phylogenomic tree showing the position of strain WL48A^T within the genus *Geodermatophilus*. Tree was reconstructed using the Bacterial Genome Tree tool (BV-BRC platform). The analysis is based on single-copy genes and the number is set as 1000. Bar represent 1 nucleotide substitution rate (Knuc) units. Black circles represent bootstrap values equal to 100. *Frankia alni* ACN14A^T was used as outgroup.

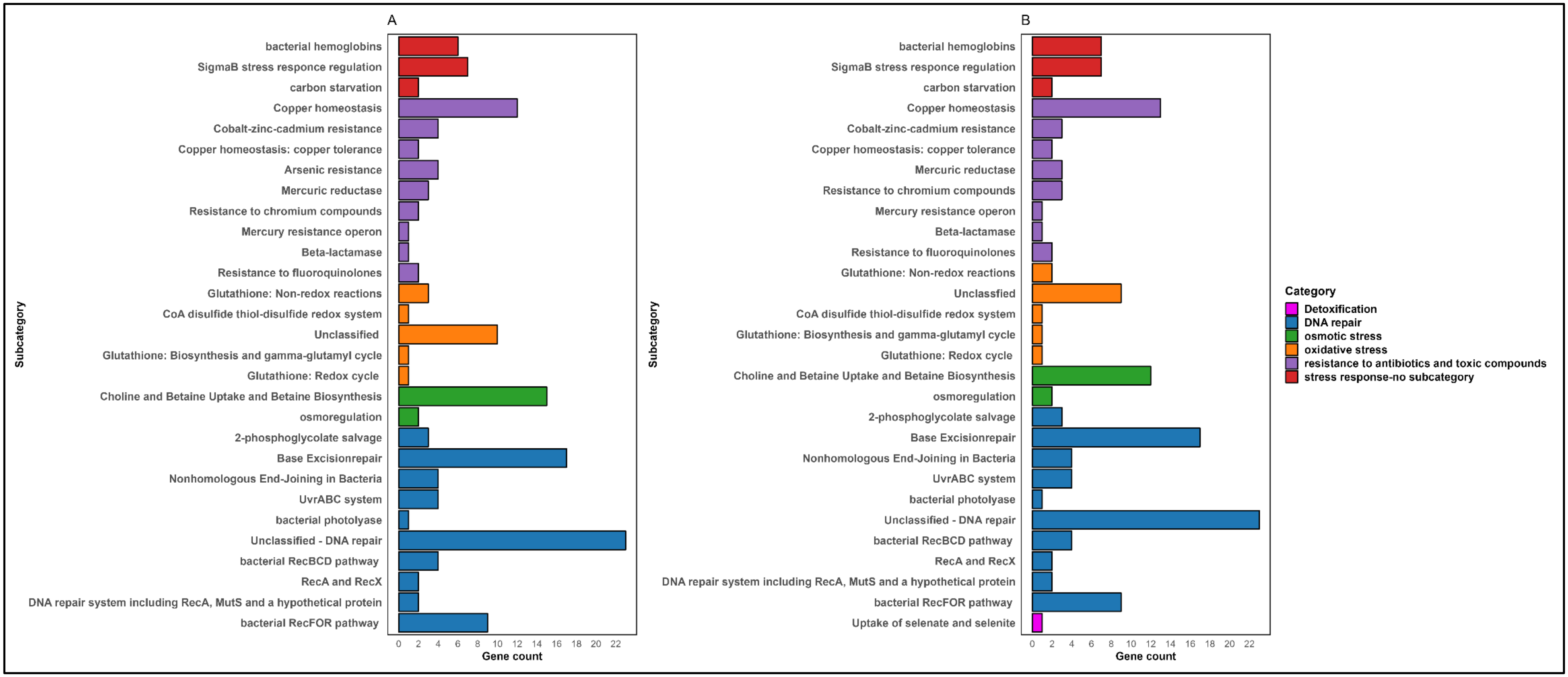

Figure S3. Barplots illustrating predictive gene functions associated with various stress responses, resistance to antibiotics and toxic compounds for (A): strain WL48A^T, and its closest phylogenomic type strain (B): *G. bullaregiensis* DSM 46841^T

Table S1. The biosynthetic gene clusters (BGCs) of strain WL48A^T identified by antiSMASH

Type	Size (kb)	Most Similar Known Cluster	Similarity
redox-cofactor	22	lankacidin C	20%
T1PKS	45	amycolamycin A/amycolamycin B	20%
betalactone	24	formicamycins A-M	6%
terpene	21	tetrocarcin A	4%
terpene	18	isorenieratene	25%
T1PKS	34	maduropeptin	18%
T3PKS	26	loseolamycin A1/loseolamycin A2	8%
ranthipeptide	21	-	-
T2PKS	16	xantholipin	12%

Table S2. The biosynthetic gene clusters (BGCs) of *G. bullaregiensis* DSM 46841^{T} identified by antiSMASH

Type	Size (kb)	Most Similar Known Cluster	Similarity
terpene	20	isorenieratene	25%
T3PKS	41	loseolamycin A1/loseolamycin A2	8%
ranthipeptide	21	-	-
T2PKS	72	formicamycins A-M	18%
terpene	21	tetrocarcin A	4%
redox-cofactor	22	lankacidin C	13%

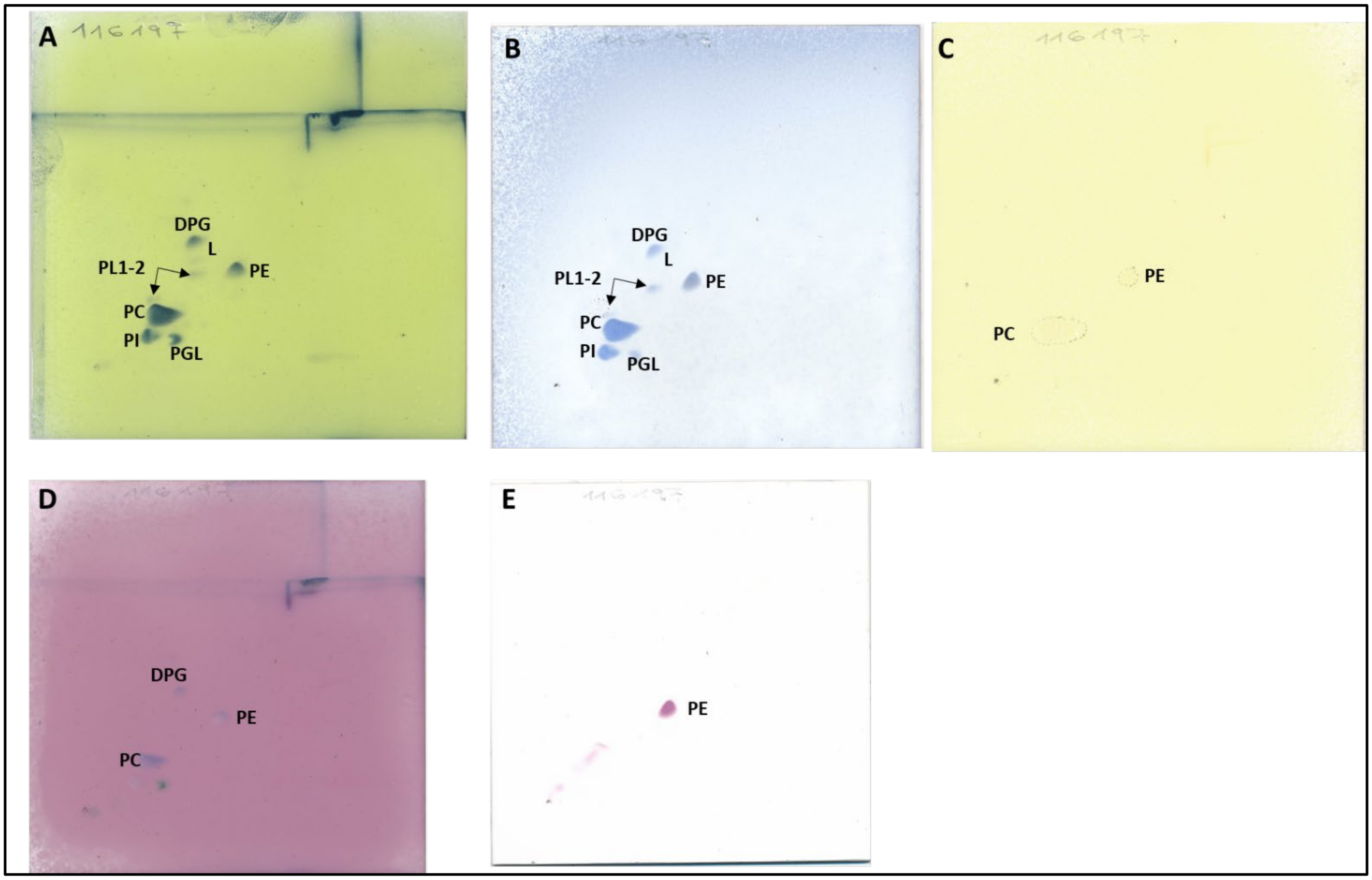

Figure S4. Two-dimensional thin layer chromatography showing the total polar lipid profile of strain WL48A^T. 30 μl extract was applied on silica HPTLC plates (Merck .05631.0001) and developed in solvent 1 (chlorophorm-methanol-water; 65:254) in the first direction followed by solvent 2 (chlorophormacetic acid-methanol-water; 80:15:12:4) in the second direction. The results were visualized by phosphomolybdic acid spray (Sigma 319279). Abbreviations: PC: phosphatidylcholine, PI: phosphatidylinositol, DPG: diphosphatidylglycerol, PE: phosphatidylethanolamine, PGL: phosphoglycolipid, Pl: phospholipids, L: unidentified lipid

Table S3. Complete cellular fatty acid profile (%) of the strain WL48A^T and *G. bullaregiensis* DSM 46841^T

Name	WL48A^T	DSM 46841^T
$C_{12:0}$	0.1	0.4
iso-$C_{13:0}$	0.1	0.2
anteiso-$C_{13:0}$	0.1	-
iso-$C_{14:0}$	0.6	1
$C_{14:0}$	1.2	0.7
iso-$C_{15:1}$ $\omega 5c$	-	0.2
iso-$C_{15:1}$ $\omega 6c$	-	1.2
iso-$C_{15:0}$	19.2	24.6
anteiso-$C_{15:0}$	5.3	2.7
$C_{15:1}$ $\omega 6c$	0.1	0.2
$C_{15:0}$	0.8	0.9
iso-$C_{16:0}$	18.9	28.2
iso-$C_{16:1}$ $\omega 6c$	0.7	-
$C_{16:1}$ $\omega 7c$	4	2.9
$C_{16:0}$	10.5	6.6
iso-$C_{17:1}$ $\omega 7c$	0.5	0.9
anteiso-$C_{17:1}$ $\omega 7c$	0.2	0.2
iso-$C_{17:0}$	5.3	6.8
anteiso-$C_{17:0}$	11.1	5.1
$C_{17:1}$ $\omega 8c$	1.2	2.3
$C_{17:0}$	5.9	6.3
iso-$C_{18:0}$	0.3	0.5
$C_{18:1}$ $\omega 9c$	3.7	2.2
$C_{18:1}$ $\omega 7c$	0.6	0.2
$C_{18:0}$	9.5	5.8
